# Supplementary material for: Recurrent integration of human papillomavirus genomes at transcriptional regulatory hubs
Source: NPJ Genom Med. 2021 Nov 30;6:101. doi: 10.1038/s41525-021-00264-y (PMC8632991; doi:10.1038/s41525-021-00264-y)
Supplement: Supplementary file 1 — Supplementary Information [file 41525_2021_264_MOESM1_ESM.pdf]

SUPPLEMENTARY INFORMATION

Recurrent Integration of Human Papillomavirus Genomes at Transcriptional Regulatory Hubs

Alix Warburton[1], Tovah E. Markowitz[2-3], Joshua P. Katz[4], James M. Pipas[4] and Alison A. McBride[1 *]

[1]*Laboratory of Viral Diseases, National Institute of Allergy and Infectious Diseases, 33 North Drive, MSC3209, National Institutes of Health, Bethesda, Maryland 20892, USA*

[2]*NIAID Collaborative Bioinformatics Resource (NCBR), National Institute of Allergy and Infectious Diseases, National Institutes of Health, Bethesda, MD, USA*

[3]*Advanced Biomedical Computational Science, Frederick National Laboratory for Cancer Research, Frederick, MD, USA*

[4]*Department of Biological Sciences, University of Pittsburgh, Pittsburgh, Pennsylvania, USA*

This document contains Supplementary Figures 1-5 and Supplementary Data Table descriptions.

Supplementary Data Tables 1-17 are included in a separate Excel file.

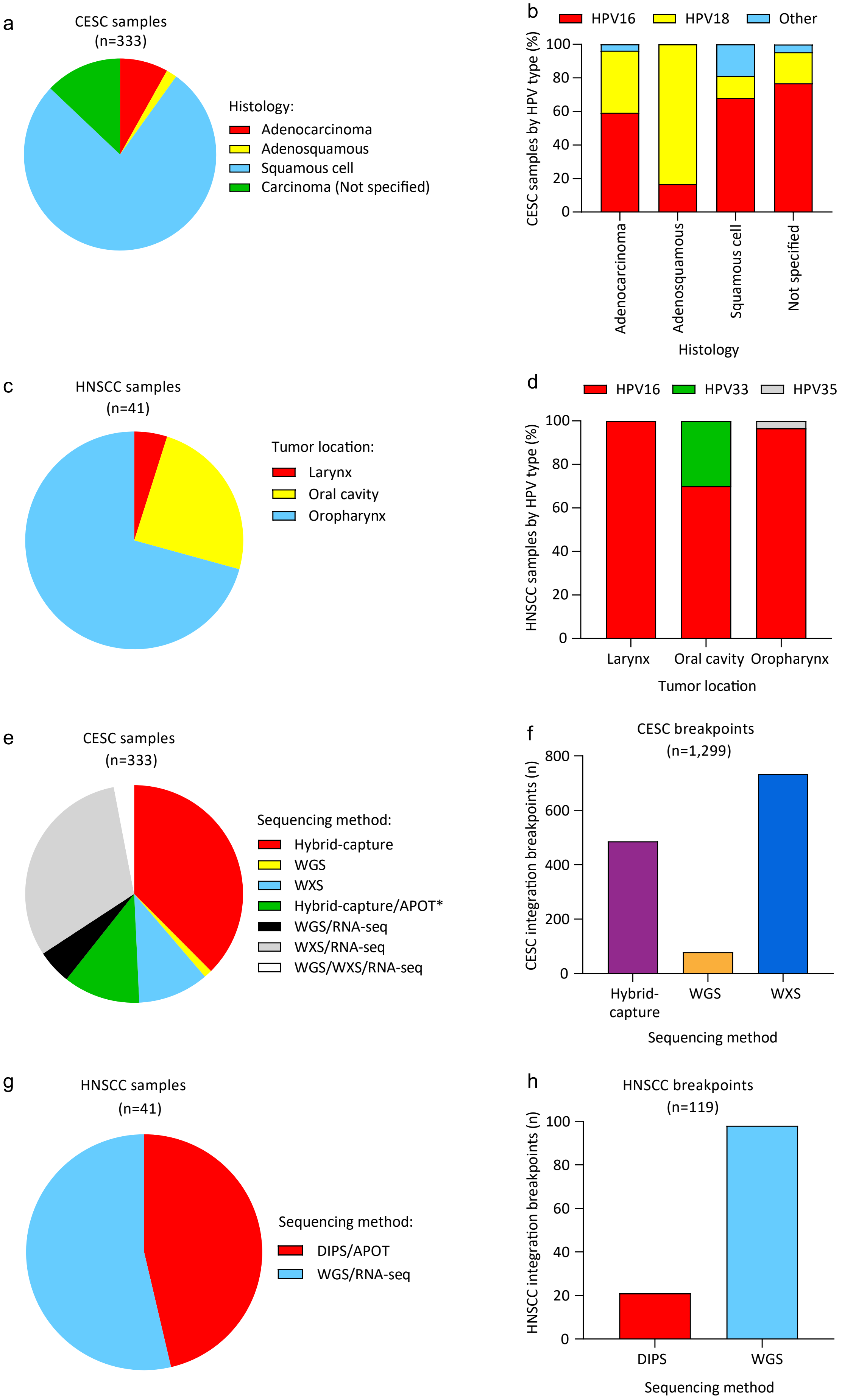

a
CESC samples
(n=333)
Histology:
Adenocarcinoma
Adenosquamous
Squamous cell
Carcinoma (Not specified)
b
HPV16
HPV18
Other
CESC samples by HPV type (%)
0
20
40
60
80
100
Adenocarcinoma
Adenosquamous
Squamous cell
Not specified
Histology
c
HNSCC samples
(n=41)
Tumor location:
Larynx
Oral cavity
Oropharynx
d
HPV16
HPV33
HPV35
HNSCC samples by HPV type (%)
0
20
40
60
80
100
Larynx
Oral cavity
Oropharynx
Tumor location
e
CESC samples
(n=333)
Sequencing method:
Hybrid-capture
WGS
WXS
Hybrid-capture/APOT*
WGS/RNA-seq
WXS/RNA-seq
WGS/WXS/RNA-seq
f
CESC breakpoints
(n=1,299)
CESC integration breakpoints (n)
0
200
400
600
800
Hybrid-capture
WGS
WXS
Sequencing method
g
HNSCC samples
(n=41)
Sequencing method:
DIPS/APOT
WGS/RNA-seq
h
HNSCC breakpoints
(n=119)
HNSCC integration breakpoints (n)
0
20
40
60
80
100
DIPS
WGS
Sequencing method

Supplementary Figure 1. Characteristics of CESC and HNSCC HPV integration datasets. (**a**), The distribution of cervical carcinomas (CESC, n=333) based on tissue histology. Squamous cell carcinomas, adenocarcinomas and adenosquamous carcinomas accounted for 77.2%, 8.1% and 1.8% of cervical samples, respectively. CESC that were not specified by histology type accounted for 12.9% of the samples. (**b**), The frequency of HPV types across the different histological subtypes. One CESC sample (*TCGA-EA-A43B*) had two integration sites of different viral types and was therefore excluded from these counts. HPV16 (67.5%) and HPV18 (17.2%) were the most frequent HPV types detected in CESC. HPV16 was the predominant viral type in squamous cell carcinomas (HPV16, 68.0%; HPV18, 13.3%; other, 18.8%) and adenocarcinomas (HPV16, 59.3%; HPV18, 37.0%; other, 3.7%), whereas HPV18 was the predominant viral type in adenosquamous carcinomas (HPV18, 83.3%; HPV16, 16.7%; other, none). Head and neck squamous cell carcinomas (n=41) included in this study were predominantly male subjects (males, 82.9%; females, 17.1%), **Supplementary Data Table 2**. (**c**), Tumors of the oropharynx, including the base of tongue and tonsils, accounted for 70.7% of samples. The remainder of tumors were from the oral cavity (24.4%) and larynx (4.9%). (**d**), The percentage distribution of HPV type by HNSCC tumor location. HPV16 was the most predominant viral type in HNSCC (90.2%) and accounted for 96.6%, 70.0% and 100% tumors of the oropharynx, oral cavity and larynx, respectively. The remainder of HNSCC samples were positive for HPV33 (7.3%) and HPV35 (2.4%) that were isolated from the oral cavity and oropharynx, respectively. (**e-h**), The distribution of samples and integration breakpoints included in this study that were grouped by sequencing method. (**e-f**), For the CESC dataset, 50.8% samples had associated transcription-based data (**e**) and all integration breakpoints were detected through next-generation sequencing technologies (hybrid-capture/WGS/WXS with probes added to capture the integrated HPV DNA sequence) (**f**). (**g**), The HNSCC dataset was limited in size as >50% samples were identified from RNA sequencing alone and did not have matched DNA sequences. (**h**), Approximately 54% HNSCC samples were processed from WGS and 46% from DIPS, and all had matched RNA data (RNA-seq and APOT, respectively). *Two samples were

also validated by RNA-seq analysis in addition to APOT. *APOT, Amplification of papillomavirus oncogene transcripts; DIPS, Detection of integrated papillomavirus sequences; WGS, Whole genome sequencing; WXS, Whole exome sequencing*.

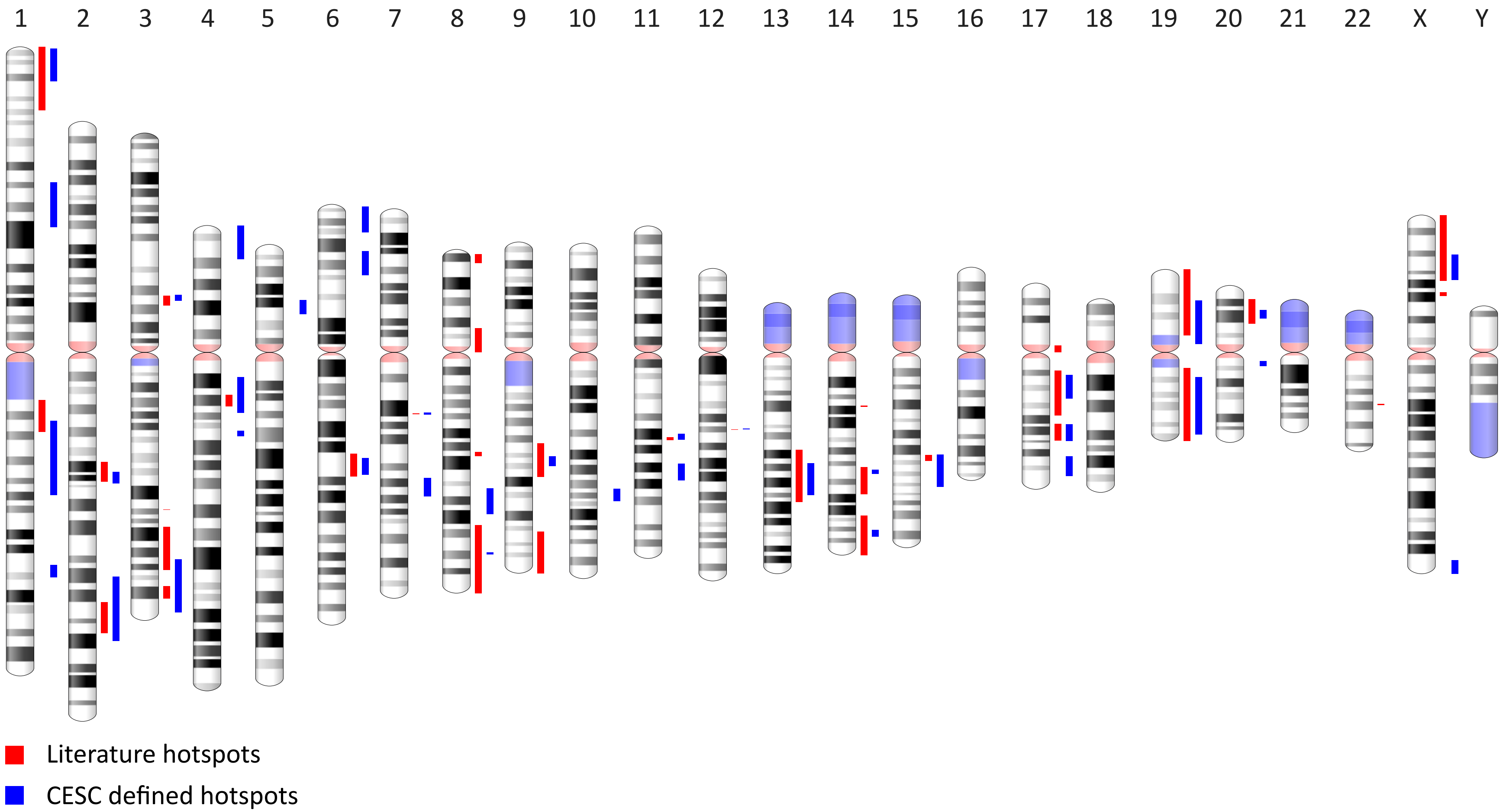

Supplementary Figure 2. Integration hotspots defined in cervical tumors. Schematic representation of integration hotspots across the human genome defined previously in the literature (red bars) and in our cervical carcinoma, CESC, dataset (blue bars).

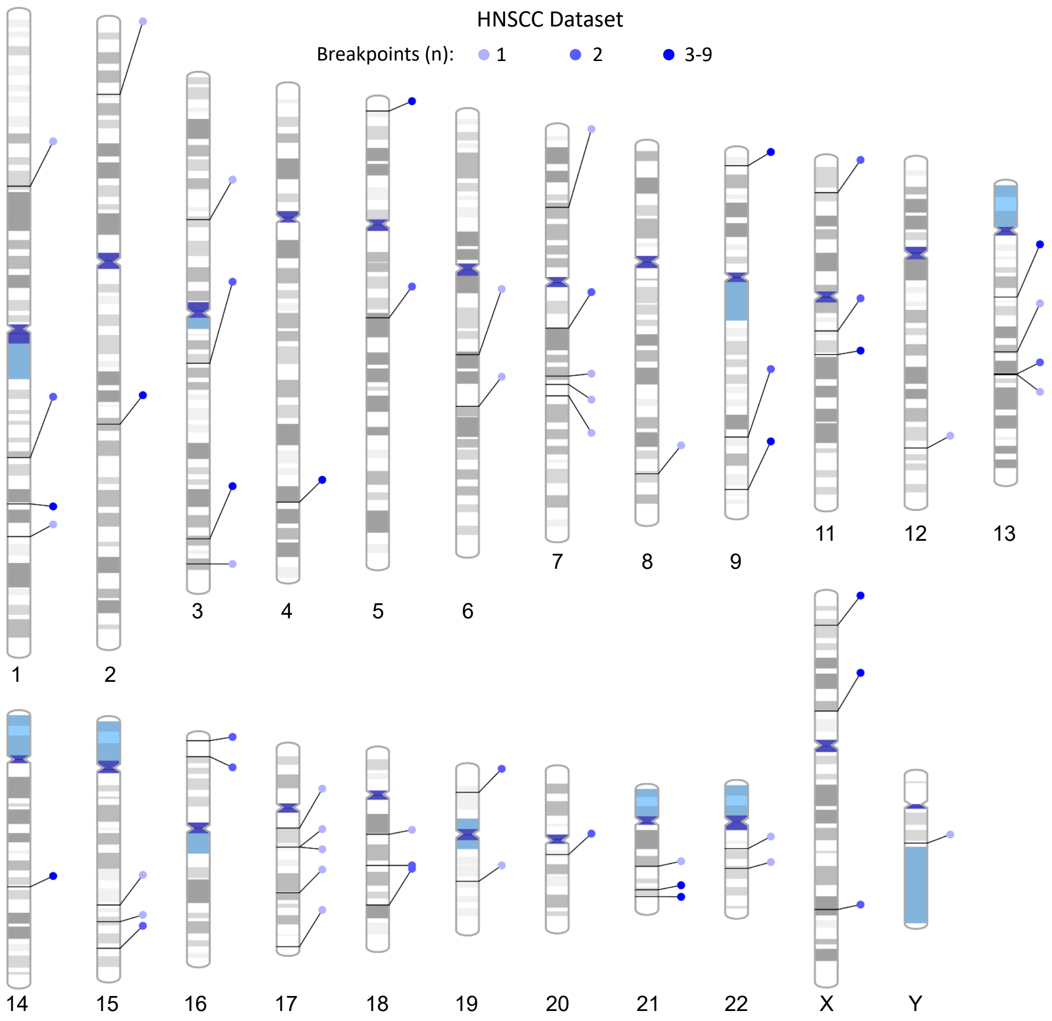

Supplementary Figure 3. Distribution of clustered breakpoints at HNSCC integration loci. Schematic representation of clustered breakpoints at HNSCC integration loci across the human genome. Lines connecting to each chromosome represent different integration loci. Blue circles represent the indicated number of breakpoints per integration locus.

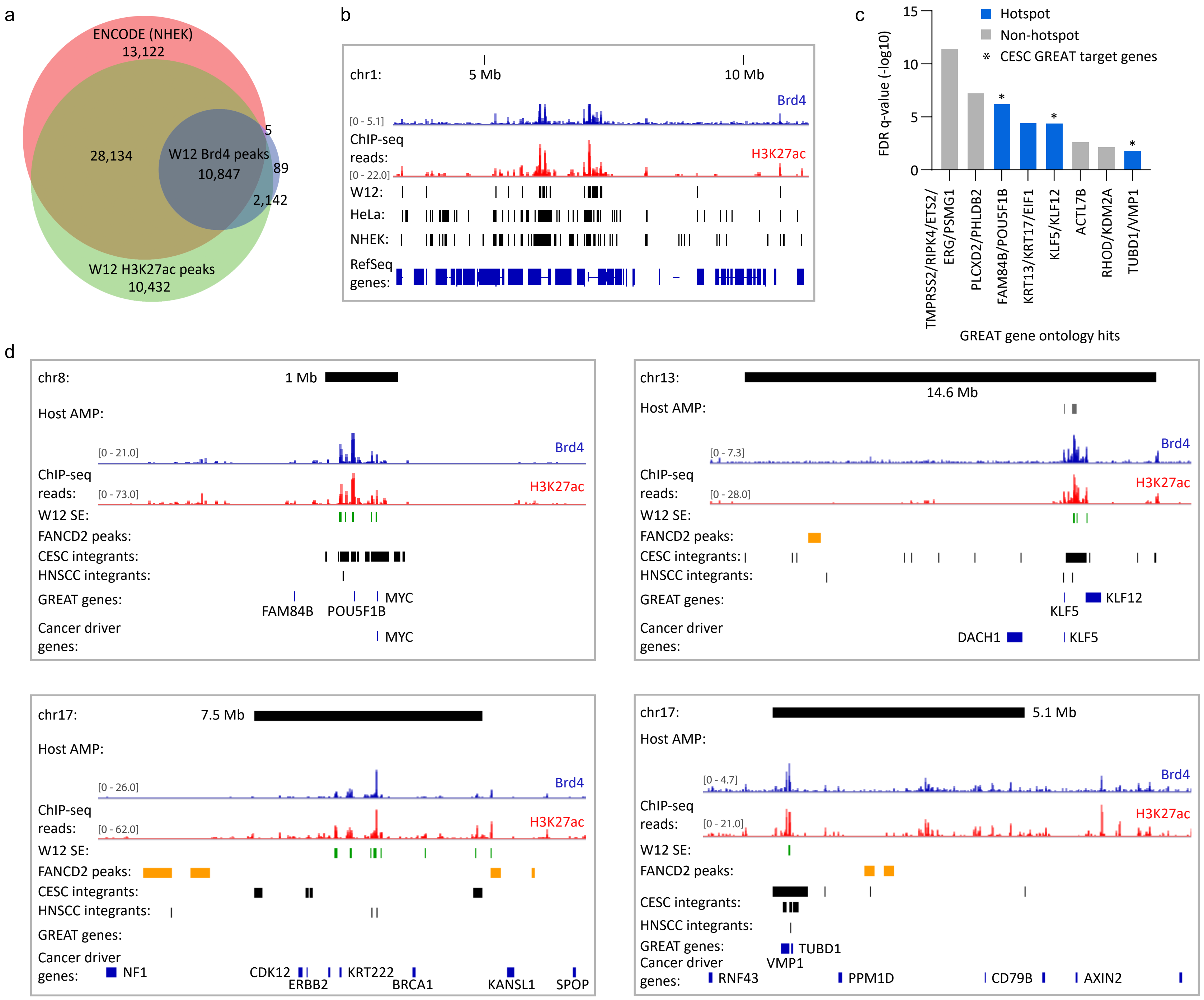
a
ENCODE (NHEK)
13,122
28,134
W12 Brd4 peaks
10,847
5
89
2,142
W12 H3K27ac peaks
10,432
b
chr1:
5 Mb
10 Mb
Brd4
[0 - 5.1]
ChIP-seq
reads:
H3K27ac
[0 - 22.0]
W12:
HeLa:
NHEK:
RefSeq
genes:
c
Hotspot
Non-hotspot
* CESC GREAT target genes
FDR q-value (-log10)
15
10
5
0
TMPRSS2/RIPK4/ETS2/
ERG/PSMG1
PLCXD2/PHLDB2
FAM84B/POU5F1B
KRT13/KRT17/EIF1
KLF5/KLF12
ACTL7B
RHOD/KDM2A
TUBD1/VMP1
GREAT gene ontology hits
d
chr8:
1 Mb
Host AMP:
[0 - 21.0]
Brd4
ChIP-seq
reads:
[0 - 73.0]
H3K27ac
W12 SE:
FANCD2 peaks:
CESC integrants:
HNSCC integrants:
GREAT genes:
MYC
FAM84B
POU5F1B
Cancer driver
genes:
MYC
chr13:
14.6 Mb
Host AMP:
[0 - 7.3]
Brd4
ChIP-seq
reads:
[0 - 28.0]
H3K27ac
W12 SE:
FANCD2 peaks:
CESC integrants:
HNSCC integrants:
GREAT genes:
KLF12
KLF5
Cancer driver
genes:
DACH1
KLF5
chr17:
7.5 Mb
Host AMP:
[0 - 26.0]
Brd4
ChIP-seq
reads:
[0 - 62.0]
H3K27ac
W12 SE:
FANCD2 peaks:
CESC integrants:
HNSCC integrants:
GREAT genes:
Cancer driver
genes:
NF1
CDK12
KRT222
ERBB2
BRCA1
KANSL1
SPOP
chr17:
5.1 Mb
Host AMP:
[0 - 4.7]
Brd4
ChIP-seq
reads:
[0 - 21.0]
H3K27ac
W12 SE:
FANCD2 peaks:
CESC integrants:
HNSCC integrants:
GREAT genes:
TUBD1
VMP1
Cancer driver
genes:
RNF43
PPM1D
CD79B
AXIN2

Supplementary Figure 4. Keratinocyte-specific enhancers mapped by Brd4 and H3K27ac ChIP-seq. (**a**), Venn diagram showing the regions of overlap between Brd4 and H3K27ac ChIP-seq signals profiled in W12 cervical keratinocytes with enhancers defined in NHEK (Normal Human Epidermal Keratinocytes) cells from ENCODE [1]. Permutation testing was used to determine the significance in overlap between W12 Brd4/H3K27ac consensus peaks and ENCODE NHEK enhancers ($p<0.0001$). (**b**), Alignment of Brd4 (blue) and H3K27ac (red) ChIP-seq signals mapped in W12 cervical keratinocytes with enhancers defined in the HeLa and NHEK ENCODE datasets. Relative ChIP-seq peak heights are indicated in square parentheses. Black bars below the ChIP-seq signal tracks represent W12 enhancers that were defined by consensus peaks for Brd4 and H3K27ac enrichment. (**c**), GREAT (Genomic Regions Enrichment of Annotations Tool) gene ontology analysis was performed using W12 enhancers that overlapped HNSCC integration breakpoints (-/+ 50 Kb flanks) as input, and compared against all W12 enhancers, to identify putative target genes associated with these cis-regulatory regions. Bars represent putative target genes plotted against their FDR (false discovery rate) adjusted p-values (q-value). Blue and grey bars represent genes that overlap integration hotspots and sites of non-recurrent integration, respectively. Enriched target genes within the same genomic locus were grouped (e.g. KLF5 and KLF12) and plotted using the most significant q-value. (**d**), Alignment of Brd4 (blue) and H3K27ac (red) ChIP-seq signals mapped in W12 cervical keratinocytes at integration hotspots (black bars; size indicated in Mb) in cervical carcinomas. Relative ChIP-seq peak heights are indicated in square parentheses. Grey bars represent amplified (AMP) host DNA in different HNSCC tumors from The Cancer Genome Atlas. Green, yellow, and black bars below the ChIP-seq signal tracks represent super-enhancers (SE) mapped in W12 subclones, FANCD2-associated fragile sites mapped in C33-A and HeLa cells and CESC/HNSCC integration loci, respectively. Genes identified from GREAT gene ontology analysis [2] and cancer driver genes [3] are indicated by blue bars.

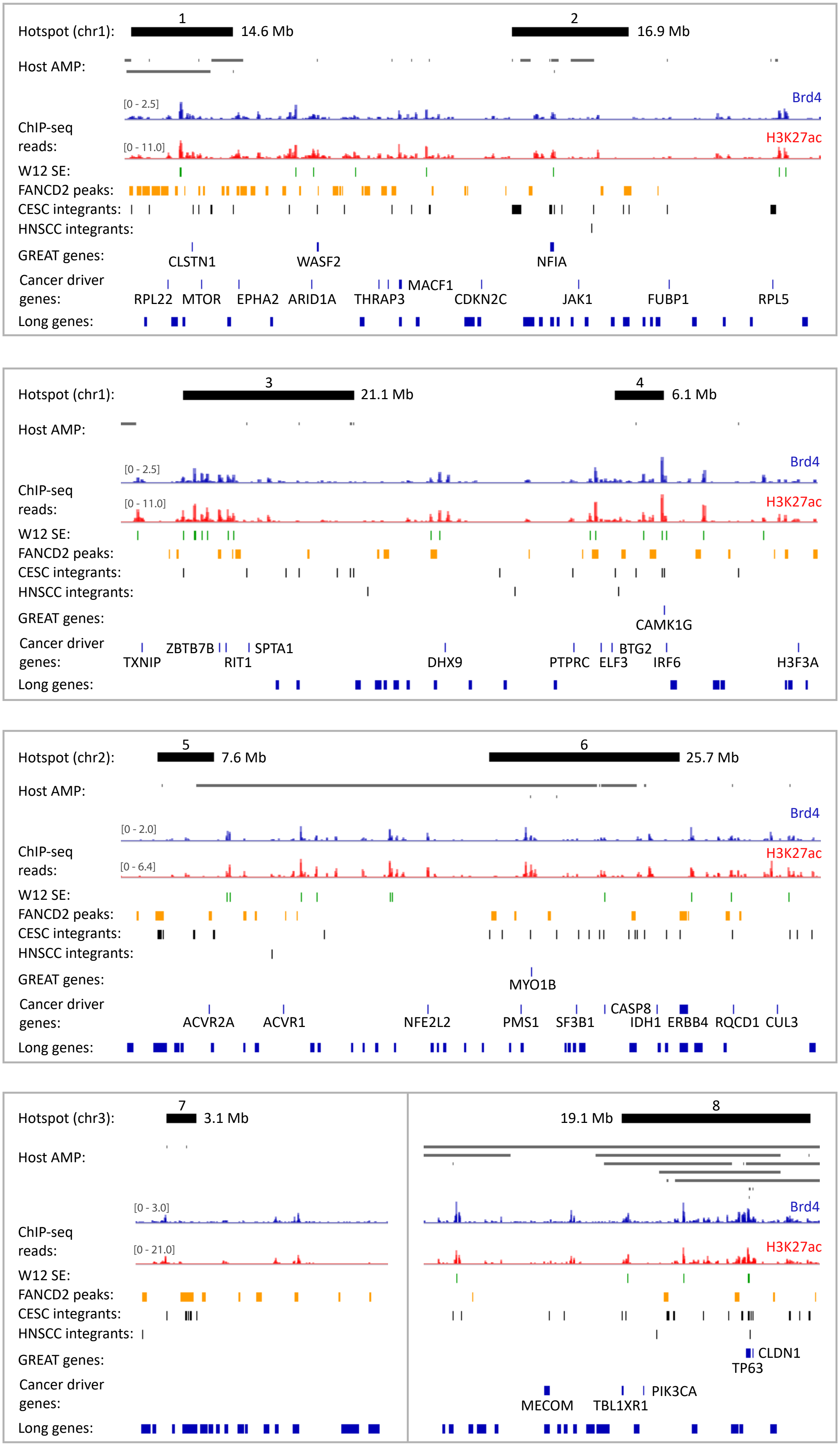
Hotspot (chr1):
1
14.6 Mb
2
16.9 Mb
Host AMP:
ChIP-seq reads:
[0 - 2.5]
Brd4
[0 - 11.0]
H3K27ac
W12 SE:
FANCD2 peaks:
CESC integrants:
HNSCC integrants:
GREAT genes:
CLSTN1
WASF2
NFIA
Cancer driver genes:
RPL22
MTOR
EPHA2
ARID1A
THRAP3
MACF1
CDKN2C
JAK1
FUBP1
RPL5
Long genes:
Hotspot (chr1):
3
21.1 Mb
4
6.1 Mb
Host AMP:
ChIP-seq reads:
[0 - 2.5]
Brd4
[0 - 11.0]
H3K27ac
W12 SE:
FANCD2 peaks:
CESC integrants:
HNSCC integrants:
GREAT genes:
CAMK1G
Cancer driver genes:
TXNIP
ZBTB7B
RIT1
SPTA1
DHX9
PTPRC
ELF3
BTG2
IRF6
H3F3A
Long genes:
Hotspot (chr2):
5
7.6 Mb
6
25.7 Mb
Host AMP:
ChIP-seq reads:
[0 - 2.0]
Brd4
[0 - 6.4]
H3K27ac
W12 SE:
FANCD2 peaks:
CESC integrants:
HNSCC integrants:
GREAT genes:
MYO1B
Cancer driver genes:
ACVR2A
ACVR1
NFE2L2
PMS1
SF3B1
CASP8
IDH1
ERBB4
RQCD1
CUL3
Long genes:
Hotspot (chr3):
7
3.1 Mb
8
19.1 Mb
Host AMP:
ChIP-seq reads:
[0 - 3.0]
[0 - 21.0]
Brd4
H3K27ac
W12 SE:
FANCD2 peaks:
CESC integrants:
HNSCC integrants:
GREAT genes:
CLDN1
TP63
Cancer driver genes:
MECOM
TBL1XR1
PIK3CA
Long genes:

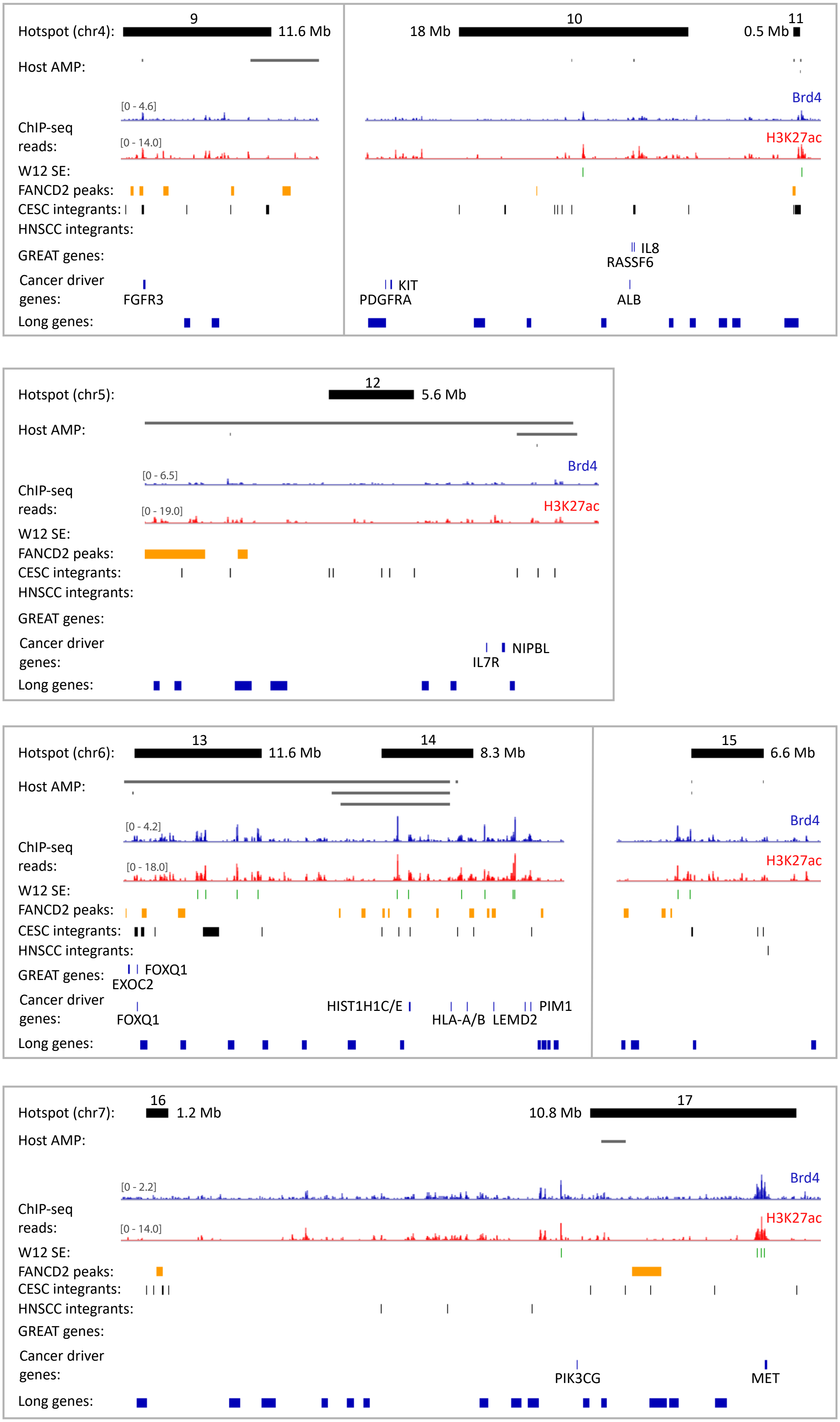

Hotspot (chr4):
9
11.6 Mb
10
18 Mb
11
0.5 Mb
Host AMP:
ChIP-seq reads:
[0 - 4.6]
[0 - 14.0]
Brd4
H3K27ac
W12 SE:
FANCD2 peaks:
CESC integrants:
HNSCC integrants:
GREAT genes:
IL8
RASSF6
Cancer driver genes:
FGFR3
KIT
PDGFRA
ALB
Long genes:
Hotspot (chr5):
12
5.6 Mb
Host AMP:
[0 - 6.5]
Brd4
ChIP-seq reads:
[0 - 19.0]
H3K27ac
W12 SE:
FANCD2 peaks:
CESC integrants:
HNSCC integrants:
GREAT genes:
Cancer driver genes:
NIPBL
IL7R
Long genes:
Hotspot (chr6):
13
11.6 Mb
14
8.3 Mb
15
6.6 Mb
Host AMP:
[0 - 4.2]
Brd4
ChIP-seq reads:
[0 - 18.0]
H3K27ac
W12 SE:
FANCD2 peaks:
CESC integrants:
HNSCC integrants:
GREAT genes:
FOXQ1
EXOC2
Cancer driver genes:
HIST1H1C/E
PIM1
FOXQ1
HLA-A/B
LEMD2
Long genes:
Hotspot (chr7):
16
1.2 Mb
10.8 Mb
17
Host AMP:
[0 - 2.2]
Brd4
ChIP-seq reads:
[0 - 14.0]
H3K27ac
W12 SE:
FANCD2 peaks:
CESC integrants:
HNSCC integrants:
GREAT genes:
Cancer driver genes:
PIK3CG
MET
Long genes:

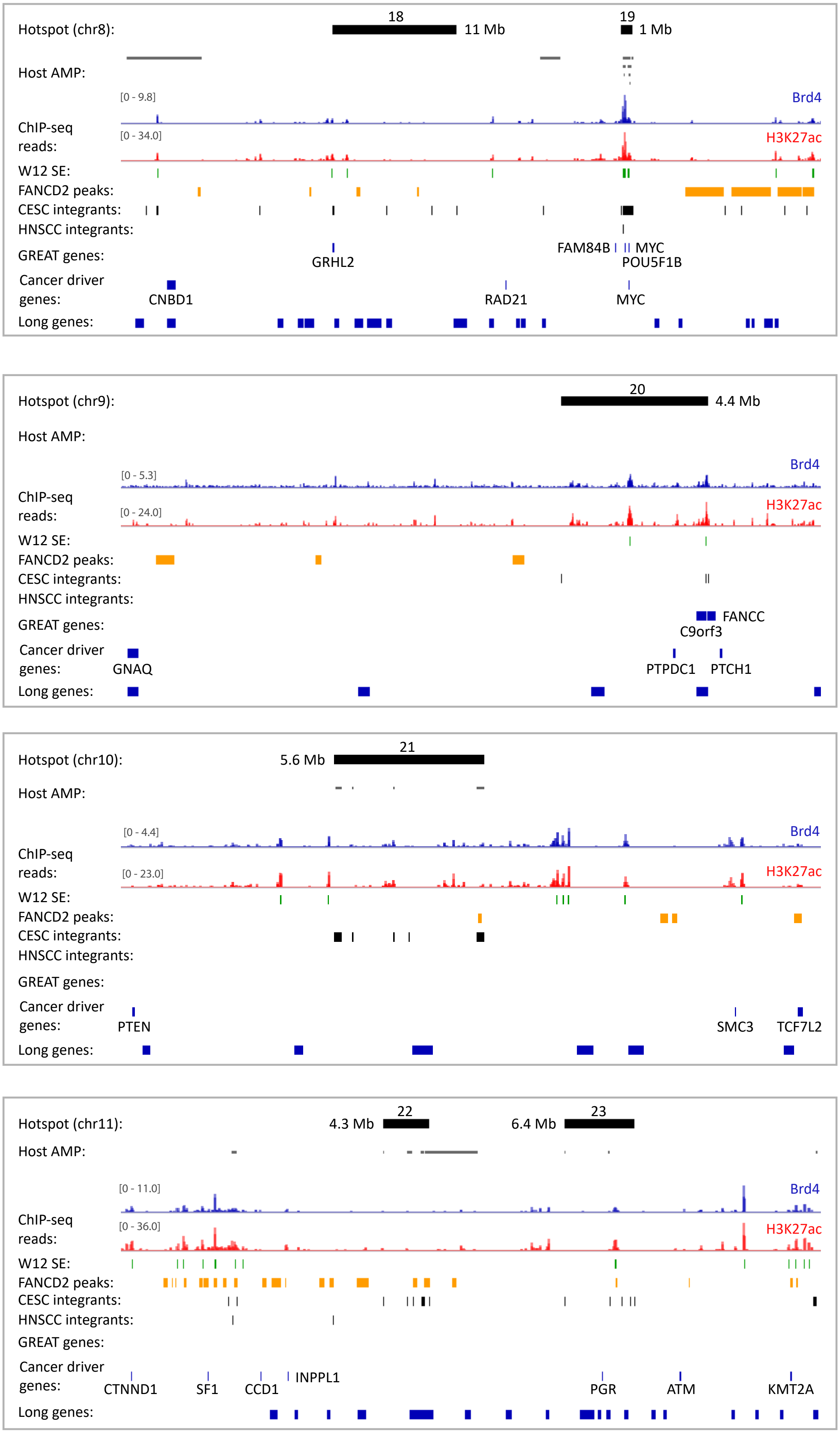
Hotspot (chr8):
18
11 Mb
19
1 Mb
Host AMP:
ChIP-seq reads:
[0 - 9.8]
Brd4
[0 - 34.0]
H3K27ac
W12 SE:
FANCD2 peaks:
CESC integrants:
HNSCC integrants:
GREAT genes:
GRHL2
FAM84B
MYC
POU5F1B
Cancer driver genes:
CNBD1
RAD21
MYC
Long genes:
Hotspot (chr9):
20
4.4 Mb
Host AMP:
ChIP-seq reads:
[0 - 5.3]
Brd4
[0 - 24.0]
H3K27ac
W12 SE:
FANCD2 peaks:
CESC integrants:
HNSCC integrants:
GREAT genes:
FANCC
C9orf3
Cancer driver genes:
GNAQ
PTPDC1
PTCH1
Long genes:
Hotspot (chr10):
5.6 Mb
21
Host AMP:
ChIP-seq reads:
[0 - 4.4]
Brd4
[0 - 23.0]
H3K27ac
W12 SE:
FANCD2 peaks:
CESC integrants:
HNSCC integrants:
GREAT genes:
Cancer driver genes:
PTEN
SMC3
TCF7L2
Long genes:
Hotspot (chr11):
4.3 Mb
22
6.4 Mb
23
Host AMP:
ChIP-seq reads:
[0 - 11.0]
Brd4
[0 - 36.0]
H3K27ac
W12 SE:
FANCD2 peaks:
CESC integrants:
HNSCC integrants:
GREAT genes:
Cancer driver genes:
CTNND1
SF1
CCD1
INPPL1
PGR
ATM
KMT2A
Long genes:

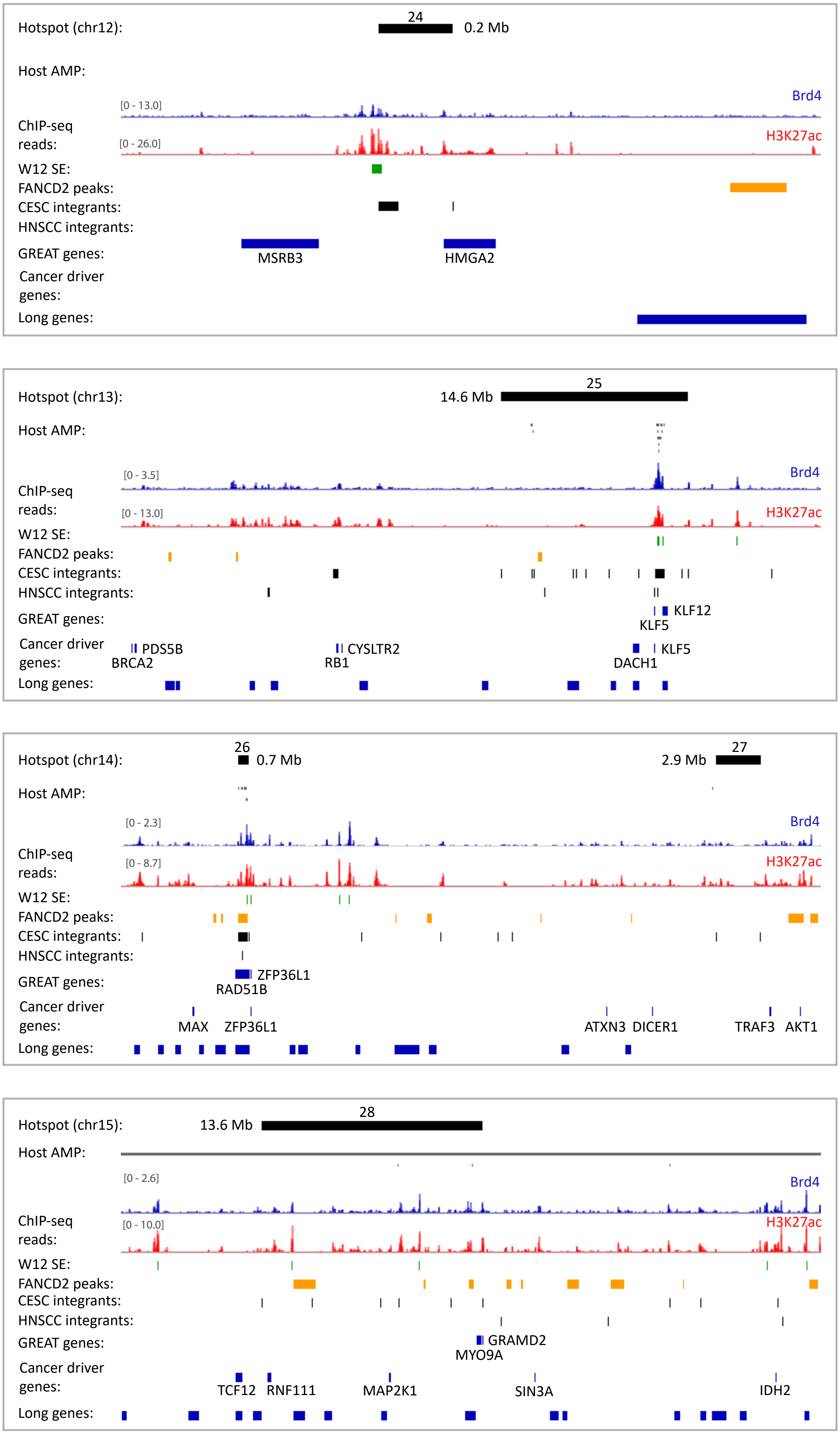

Hotspot (chr12):
24
0.2 Mb
Host AMP:
ChIP-seq reads:
[0 - 13.0]
Brd4
[0 - 26.0]
H3K27ac
W12 SE:
FANCD2 peaks:
CESC integrants:
HNSCC integrants:
GREAT genes:
MSRB3
HMGA2
Cancer driver genes:
Long genes:
Hotspot (chr13):
25
14.6 Mb
Host AMP:
ChIP-seq reads:
[0 - 3.5]
Brd4
[0 - 13.0]
H3K27ac
W12 SE:
FANCD2 peaks:
CESC integrants:
HNSCC integrants:
GREAT genes:
KLF12
KLF5
Cancer driver genes:
PDS5B
BRCA2
CYSLTR2
RB1
KLF5
DACH1
Long genes:
Hotspot (chr14):
26
0.7 Mb
27
2.9 Mb
Host AMP:
ChIP-seq reads:
[0 - 2.3]
Brd4
[0 - 8.7]
H3K27ac
W12 SE:
FANCD2 peaks:
CESC integrants:
HNSCC integrants:
GREAT genes:
ZFP36L1
RAD51B
Cancer driver genes:
MAX
ZFP36L1
ATXN3
DICER1
TRAF3
AKT1
Long genes:
Hotspot (chr15):
28
13.6 Mb
Host AMP:
ChIP-seq reads:
[0 - 2.6]
Brd4
[0 - 10.0]
H3K27ac
W12 SE:
FANCD2 peaks:
CESC integrants:
HNSCC integrants:
GREAT genes:
GRAMD2
MYO9A
Cancer driver genes:
TCF12
RNF111
MAP2K1
SIN3A
IDH2
Long genes:

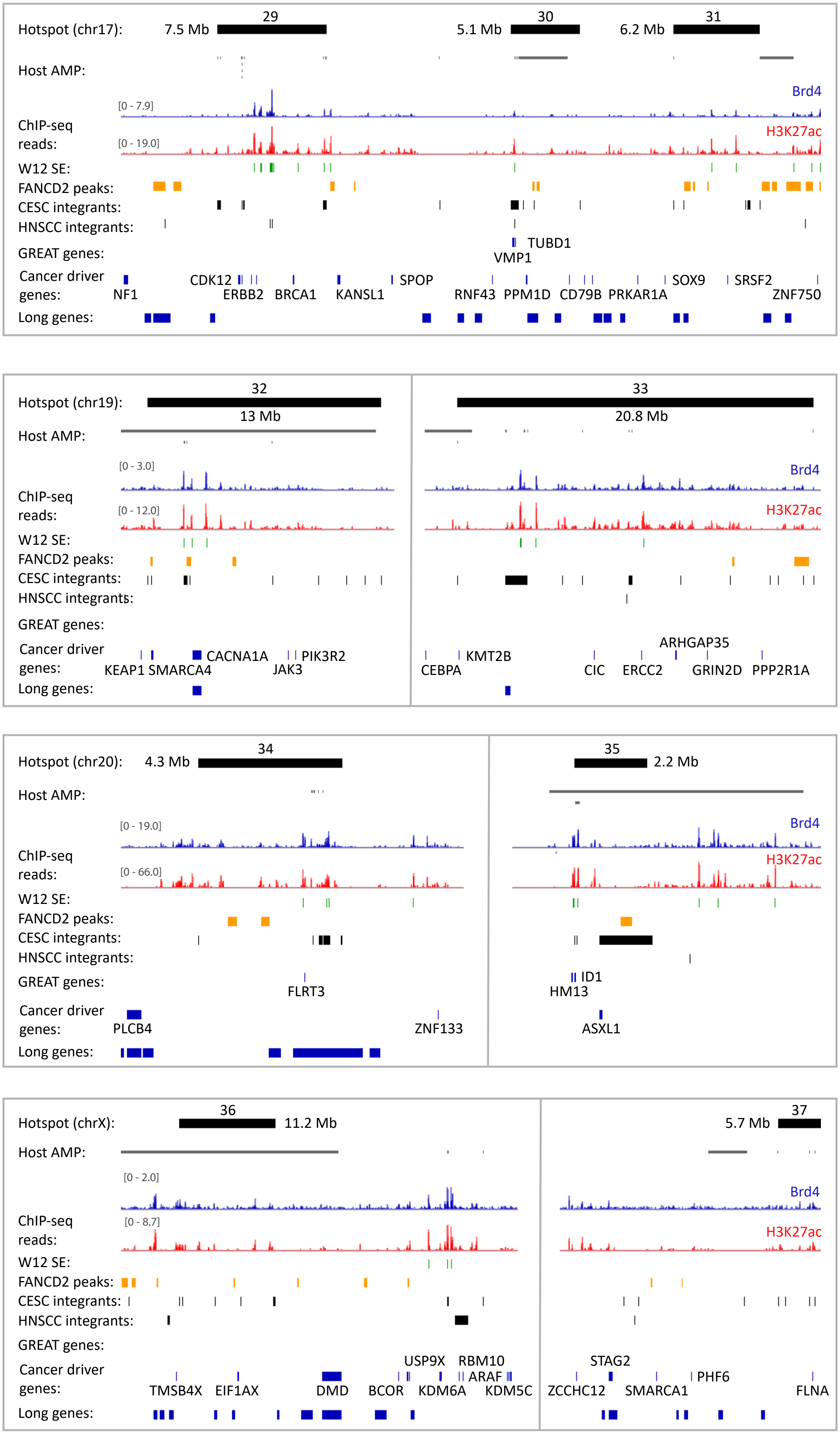

Hotspot (chr17):
29
30
31
7.5 Mb
5.1 Mb
6.2 Mb
Host AMP:
[0 - 7.9]
Brd4
ChIP-seq reads:
[0 - 19.0]
H3K27ac
W12 SE:
FANCD2 peaks:
CESC integrants:
HNSCC integrants:
GREAT genes:
TUBD1
VMP1
Cancer driver genes:
NF1
CDK12
ERBB2
BRCA1
KANSL1
SPOP
RNF43
PPM1D
CD79B
PRKAR1A
SOX9
SRSF2
ZNF750
Long genes:
Hotspot (chr19):
32
13 Mb
33
20.8 Mb
Host AMP:
[0 - 3.0]
Brd4
ChIP-seq reads:
[0 - 12.0]
H3K27ac
W12 SE:
FANCD2 peaks:
CESC integrants:
HNSCC integrants:
GREAT genes:
Cancer driver genes:
KEAP1
SMARCA4
CACNA1A
JAK3
PIK3R2
CEBPA
KMT2B
CIC
ERCC2
ARHGAP35
GRIN2D
PPP2R1A
Long genes:
Hotspot (chr20):
34
4.3 Mb
35
2.2 Mb
Host AMP:
[0 - 19.0]
Brd4
ChIP-seq reads:
[0 - 66.0]
H3K27ac
W12 SE:
FANCD2 peaks:
CESC integrants:
HNSCC integrants:
GREAT genes:
FLRT3
ID1
HM13
Cancer driver genes:
PLCB4
ZNF133
ASXL1
Long genes:
Hotspot (chrX):
36
11.2 Mb
37
5.7 Mb
Host AMP:
[0 - 2.0]
Brd4
ChIP-seq reads:
[0 - 8.7]
H3K27ac
W12 SE:
FANCD2 peaks:
CESC integrants:
HNSCC integrants:
GREAT genes:
Cancer driver genes:
TMSB4X
EIF1AX
DMD
BCOR
USP9X
KDM6A
RBM10
ARAF
KDM5C
ZCCHC12
STAG2
SMARCA1
PHF6
FLNA
Long genes:

Supplementary Figure 5. Genomic landscape of integration hotspots. Alignment of Brd4 (blue) and H3K27ac (red) ChIP-seq signals mapped in W12 cervical keratinocytes at integration hotspots (top black bars; size indicated in Mb) in cervical carcinomas. Relative ChIP-seq peak heights are indicated in square parentheses. Numbers above each integration hotspot indicates the hotspot ID referenced in **Supplementary Data Table 4**. Grey bars represent amplified (AMP) host DNA in different CESC tumors from The Cancer Genome Atlas. Green, yellow, and black bars below the ChIP-seq signal tracks represent super-enhancers (SE) mapped in W12 subclones, FANCD2-associated fragile sites mapped in C33-A and HeLa cells and CESC/HNSCC integration loci, respectively. Long protein-coding genes (>0.3 Mb in length), genes identified from GREAT gene ontology analysis [2] and cancer driver genes [3] are indicated by blue bars. Each integration hotspot is further characterized in **Supplementary Data Table 4**.

SUPPLEMENTARY DATA TABLE DESCRIPTIONS

Supplementary Data Table 1. CESC and HNSCC datasets included in our study. Cases represent the number (n) of patient samples with integrated HPV genomes detected through DNA and/or RNA sequencing methods. For hybrid-capture method, only integration breakpoints that were validated through Sanger sequencing were included in this study. Somatic copy number alteration (SCNA) datasets for CESC and HNSCC TCGA samples were downloaded from the Broad Institute, http://firebrowse.org/ [4,5].

Supplementary Data Table 2. CESC integration breakpoints (n=1,299) included in this study. CESC HPV integration sites (Patient ID, Histology, HPV type, HPV breakpoints, chromosome, integration breakpoint, integration ID, number of breakpoints per integration locus, detection method used for calling integration breakpoints, PubMed ID and author of study, target gene disrupted by integration, transcription status based on detection of viral-host fusion transcripts, SCNA status, SCNA mean (values are log2(copy number/2) unless otherwise stated), distance of integration breakpoint from SCNA, SCNA position and size, number of SNP Array 6.0 probes spanning the SCNA). Only integration breakpoints detected through DNA sequencing were used for overlap analyses.

Supplementary Data Table 3. HNSCC integration breakpoints (n=119) included in this study. HNSC HPV integration sites (Patient ID, gender, Histology, tumor location, HPV type, HPV breakpoints, chromosome, integration breakpoint, integration ID, number of breakpoints per integration locus, detection method used for calling integration breakpoints, PubMed ID and author of study, target gene disrupted by integration, transcription status based on detection of viral-host fusion transcripts, SCNA status, SCNA mean (values are log2(copy number/2) unless otherwise stated), distance of integration breakpoint from SCNA, SCNA position and size, number of SNP Array 6.0 probes spanning the SCNA, patient age, AJCC stage of tumor, smoking status). Only integration breakpoints detected through DNA sequencing were used for overlap analyses.

Supplementary Data Table 4. Integration hotspots defined in CESC

Supplementary Data Table 5. Integration hotspots defined in the literature. Overlapping genomic intervals for hotspots identified from the literature were merged into a single genomic interval using the MergeBED tool (column *Merged hotspot position*) for comparison to hotspots defined in our CESC dataset (**Supplementary Figure 2**).

Supplementary Data Table 6. CESC integrations with associated SCNA included in this study. Samples that had associated host genome amplification data were classified based on the somatic copy number alteration (SCNA) status at the site of integration. Normal, normal genomic profile; AMP, amplification; DEL, deletion; DEL (excluded; overlapping deletion), these likely reflect the alternative chromosome as sequencing of the chimeric viral-host junction was available for the associated HPV insertion sites. SCNA mean values are log2(copy number/2) unless otherwise stated. Overlap of integration sites with integration hotspots are indicated.

Supplementary Data Table 7. HNSCC integrations with associated SCNA included in this study. Samples that had associated host genome amplification data were classified based on the somatic copy number alteration (SCNA) status at the site of integration. Normal, normal genomic profile; AMP, amplification; DEL, deletion; DEL (excluded; overlapping deletion), these likely reflect the alternative chromosome as sequencing of the chimeric viral-host junction was available for the associated HPV insertion sites. SCNA mean values are log2(copy number/2) unless otherwise stated.

Supplementary Data Table 8. FANCD2-associated fragile sites mapped in C33-A cervical carcinoma cells. C33-A FANCD2 peaks identified through ChIP-seq, filtered by -log10 q-value >10, combined with FANCD2 ChIP-ChIP peaks previously reported in C33-A cells [6].

Supplementary Data Table 9. FANCD2-associated fragile sites mapped in HeLa cervical carcinoma cells. HeLa FANCD2 peaks identified through ChIP-seq, filtered by -log10 q-value >10.

Supplementary Data Table 10. FANCD2-associated fragile sites mapped in C33-A and HeLa cervical carcinoma cells. C33-A FANCD2 peaks identified through ChIP-ChIP and ChIP-seq (**Supplementary Data Table 8**) were combined with HeLa ChIP-seq peaks (**Supplementary Data Table 9**). Overlapping FANCD2 peaks from the C33-A and HeLa datasets were merged using the MergeBED tool (merging based on nearby peaks was set to 0 bp).

Supplementary Data Table 11. Aphidicolin-induced common fragile sites from the HGNC Database. FRA-regions were downloaded from the HUGO Gene Nomenclature Committee (HGNC) database, https://www.genenames.org/download/custom/. Bold font indicates FRA regions that overlap with FANCD2-enriched regions listed in **Supplementary Data Table 10**. A total of 43/77 (55.8%) FRA regions overlap with FANCD2-enriched regions profiled in C33-A and HeLa cells.

Supplementary Data Table 12. Mitotic DNA synthesis (MDS) regions that overlap with FANCD2 peaks. MDS regions profiled in HeLa cells [7,8] were compiled and overlapping regions merged using the MergeBED tool (merging based on nearby peaks was set to 0 bp). A total of 112/232 (48.3%) MDS regions overlapped with FANCD2-enriched regions profiled in C33-A and HeLa cells.

Supplementary Data Table 13. FANCD2-enriched regions that overlap long genes. The Gencode Release 19 human reference genome (GRCh37) was filtered to identify protein-coding genes longer than or equal to 0.3 Mb, including UTRs. A total of 185/782 (23.7%) long genes overlapped with FANCD2-enriched regions profiled in C33-A and HeLa cells and are indicated in bold font. Genes that are transcriptionally active in C33-A and/or HeLa cells were determined from RNA-seq ([6] and Expression Atlas, accessed September 2020, https://www.ebi.ac.uk/gxa/home) and are indicated in blue font. 121/185 (65.4%) long genes that overlapped with FANCD2-enriched regions are expressed in C33-A and/or HeLa cells.

Supplementary Data Table 14. Brd4/H3K27ac consensus enhancer peaks profiled in W12 subclones. Brd4 and H3K27ac peaks were identified through ChIP-seq in HPV16-positive W12 cervical keratinocyte

subclones (20831, 20861, 20862 and 20863). Consensus peaks for H3K27ac were identified across the four W12 subclones. Enhancers were defined as the overlapping genomic intervals between H3K27ac consensus peaks and Brd4 peaks.

Supplementary Data Table 15. Top 20 biological processes associated with enhancers that overlap integration breakpoints in CESC and HNSCC

Supplementary Data Table 16. Super-enhancers defined in W12 cervical keratinocytes

Supplementary Data Table 17. Cancer driver genes within 1 Mb of super-enhancers that overlap integration loci

REFERENCES

1 Consortium, E. P. *et al.* Expanded encyclopaedias of DNA elements in the human and mouse genomes. *Nature* **583**, 699-710, doi:10.1038/s41586-020-2493-4 (2020).

2 McLean, C. Y. *et al.* GREAT improves functional interpretation of cis-regulatory regions. *Nat Biotechnol* **28**, 495-501, doi:10.1038/nbt.1630 (2010).

3 Bailey, M. H. *et al.* Comprehensive Characterization of Cancer Driver Genes and Mutations. *Cell* **173**, 371-385.e318, doi:https://doi.org/10.1016/j.cell.2018.02.060 (2018).

4 Harvard, B. I. o. M. a. Broad Institute TCGA Genome Data Analysis Center (2016): SNP6 Copy number analysis (GISTIC2); Cervical Squamous Cell Carcinoma and Endocervical Adenocarcinoma. doi:doi:10.7908/C16D5SCD (2016).

5 Harvard, B. I. o. M. a. Broad Institute TCGA Genome Data Analysis Center (2016): SNP6 Copy number analysis (GISTIC2); Head and Neck Squamous Cell Carcinoma. . doi:doi:10.7908/C1V987FP (2016).

6 Jang, M. K., Shen, K. & McBride, A. A. Papillomavirus genomes associate with BRD4 to replicate at fragile sites in the host genome. *PLoS Pathog* **10**, e1004117, doi:10.1371/journal.ppat.1004117 (2014).

7 Ji, F. *et al.* Genome-wide high-resolution mapping of mitotic DNA synthesis sites and common fragile sites by direct sequencing. *Cell Res*, doi:10.1038/s41422-020-0357-y (2020).

8 Macheret, M. *et al.* High-resolution mapping of mitotic DNA synthesis regions and common fragile sites in the human genome through direct sequencing. *Cell Res*, doi:10.1038/s41422-020-0358-x (2020).
