# Supplementary material for: Recurrent integration of human papillomavirus genomes at transcriptional regulatory hubs
Source: NPJ Genom Med. 2021 Nov 30;6:101. doi: 10.1038/s41525-021-00264-y (PMC8632991; doi:10.1038/s41525-021-00264-y)
Supplement: Supplementary file 3 — Reporting Summary [file 41525_2021_264_MOESM3_ESM.pdf]

nature portfolio

Corresponding author(s): Alison McBride

Last updated by author(s): Oct 22, 2021

Reporting Summary

Nature Portfolio wishes to improve the reproducibility of the work that we publish. This form provides structure for consistency and transparency in reporting. For further information on Nature Portfolio policies, see our Editorial Policies and the Editorial Policy Checklist.

Statistics

For all statistical analyses, confirm that the following items are present in the figure legend, table legend, main text, or Methods section.

n/a	Confirmed	
☐	☒	The exact sample size (*n*) for each experimental group/condition, given as a discrete number and unit of measurement
☐	☒	A statement on whether measurements were taken from distinct samples or whether the same sample was measured repeatedly
☐	☒	The statistical test(s) used AND whether they are one- or two-sided<br>*Only common tests should be described solely by name; describe more complex techniques in the Methods section.*
☒	☐	A description of all covariates tested
☐	☒	A description of any assumptions or corrections, such as tests of normality and adjustment for multiple comparisons
☐	☒	A full description of the statistical parameters including central tendency (e.g. means) or other basic estimates (e.g. regression coefficient) AND variation (e.g. standard deviation) or associated estimates of uncertainty (e.g. confidence intervals)
☐	☒	For null hypothesis testing, the test statistic (e.g. *F*, *t*, *r*) with confidence intervals, effect sizes, degrees of freedom and *P* value noted<br>*Give P values as exact values whenever suitable.*
☒	☐	For Bayesian analysis, information on the choice of priors and Markov chain Monte Carlo settings
☒	☐	For hierarchical and complex designs, identification of the appropriate level for tests and full reporting of outcomes
☒	☐	Estimates of effect sizes (e.g. Cohen's *d*, Pearson's *r*), indicating how they were calculated

Our web collection on statistics for biologists contains articles on many of the points above.

Software and code

Policy information about availability of computer code

Data collection	No software was used for data collection.
Data analysis	prinseq-lite.pl version 0.20.2, Bowtie 2, BLASTN, SummonChimera, Cutadapt version 1.18, BWA version 0.7.17, SamToFastq, https://broadinstitute.github.io/picard/, SAMtools version 1.6, Picard MarkDuplicates, Phantompeakqualtools version 2.0, SICER version 1.1, macs version 2.1.1, bedtools MergeBED, regioneR, DiffBind, http://genome.ucsc.edu/cgi-bin/hgLiftOver, Rank Ordering of Super-Enhancers (ROSE) tool

For manuscripts utilizing custom algorithms or software that are central to the research but not yet described in published literature, software must be made available to editors and reviewers. We strongly encourage code deposition in a community repository (e.g. GitHub). See the Nature Portfolio guidelines for submitting code & software for further information.

Data

Policy information about availability of data

All manuscripts must include a data availability statement. This statement should provide the following information, where applicable:

- Accession codes, unique identifiers, or web links for publicly available datasets
- A description of any restrictions on data availability
- For clinical datasets or third party data, please ensure that the statement adheres to our policy

The data discussed in this publication have been deposited in NCBI's Gene Expression Omnibus 105 and are accessible through GEO Series accession number GSE183048 (https://www.ncbi.nlm.nih.gov/geo/query/acc.cgi?acc=%20GSE183048).

Field-specific reporting

Please select the one below that is the best fit for your research. If you are not sure, read the appropriate sections before making your selection.

☒ Life sciences ☐ Behavioural & social sciences ☐ Ecological, evolutionary & environmental sciences

For a reference copy of the document with all sections, see nature.com/documents/nr-reporting-summary-flat.pdf

Life sciences study design

All studies must disclose on these points even when the disclosure is negative.

Sample size	This study is a meta-analysis and so no sample size calculation was determined.
Data exclusions	Integration breakpoints reported from hybrid-capture alone were excluded from our meta-analysis. The use of hybrid-capture technologies for detection of viral integration sites has been reported to give high rates of false positives, and so insertion breakpoints identified by this method were only included if they were validated by other means, such as Sanger sequencing.
Replication	Brd4 and H3K27ac ChIP-seq experiments included biological replicates and called peaks were reproducible between the replicates, as assessed through a Jaccard score.
Randomization	N/A
Blinding	N/A

Reporting for specific materials, systems and methods

We require information from authors about some types of materials, experimental systems and methods used in many studies. Here, indicate whether each material, system or method listed is relevant to your study. If you are not sure if a list item applies to your research, read the appropriate section before selecting a response.

Materials & experimental systems

n/a	Involved in the study	
☐	☒	Antibodies
☐	☒	Eukaryotic cell lines
☒	☐	Palaeontology and archaeology
☒	☐	Animals and other organisms
☒	☐	Human research participants
☒	☐	Clinical data
☒	☐	Dual use research of concern

Methods

n/a	Involved in the study	
☐	☒	ChIP-seq
☒	☐	Flow cytometry
☒	☐	MRI-based neuroimaging

Antibodies

Antibodies used	FANCD2 (Bethyl, A302-174A, lot 1); Brd4 (Bethyl Laboratories A301-985A, lot 5); H3K27ac (Millipore 07–360, lot 2762292).
Validation	We have previously validated and published the use of the FANCD2 A302-174A antibody for ChIP-chip (https://doi.org/10.1371/journal.ppat.1004117) and the Brd4 A301-985A and H3K27ac 07–360 antibodies for ChIP-seq (https://doi.org/10.1371/journal.pgen.1007179). Brd4 A301-985A has been used by others for ChIP-seq (https://doi.org/10.1073/pnas.1315023111, https://doi.org/10.1038/s41591-018-0158-8, https://doi.org/10.1186/s13072-019-0286-5); H3K27ac 07-360 has been validated by Sigma Millipore for ChIP-seq (https://www.emdmillipore.com/US/en/product/Anti-acetyl-Histone-H3-Lys27-Antibody,MM_NF-07-360).

Eukaryotic cell lines

Policy information about cell lines

Cell line source(s)	C33-A and HeLa cells were purchased from ATCC; W12 cells were a gift from Dr. Paul Lambert, McArdle Laboratory for Cancer Research, WI, USA
Authentication	C33-A and HeLa cells were purchased from ATCC and have a certificate of authentication; W12 cells were karyotyped
Mycoplasma contamination	All cell lines tested negative for mycoplasma contamination
Commonly misidentified lines (See ICLAC register)	N/A

ChIP-seq

Data deposition

☒ Confirm that both raw and final processed data have been deposited in a public database such as GEO.

☒ Confirm that you have deposited or provided access to graph files (e.g. BED files) for the called peaks.

Data access links<br>*May remain private before publication.*	https://www.ncbi.nlm.nih.gov/geo/query/acc.cgi?acc=%20GSE183048
Files in database submission	GSM5550295; GSM5550296; GSM5550297; GSM5550298; GSM5550299; GSM5550300; GSM5550301; GSM5550302; GSM5550303; GSM5550304; GSM5550305; GSM5550306; GSM5550307; GSM5550308; GSM5550309; GSM5550310; GSM5550311; GSM5550312; GSM5550313; GSM5550314; GSM5550315; GSM5550316; GSM5550317; GSM5550318
Genome browser session<br>(e.g. UCSC)	N/A

Methodology

Replicates	For FANCD2 ChIP-seq, ChIP DNA from two biological replicates were pooled for C33-A and HeLa cells and compared to input controls. Overlapping peaks called between C33-A and HeLa cells were compared using regioneR (p<0.0001). For Brd4 and H3K27ac ChIP-seq, ChIP DNA from two biological replicates were sequenced for four W12 subclones (20831, 20861, 20862 and 20863). Biological replicates were compared using a Jaccard similarity coefficient.
Sequencing depth	ChIP and input DNA samples were subjected to 2 x 150 bp paired-end read sequencing to an average read depth (uniquely mapped reads) of >14 million reads per sample for FANCD2 ChIPs, >25 million reads for Brd4 ChIPs, >27 million reads for H3K27ac ChIPs and >81 million reads for input DNA controls. Average fragment lengths were 308 bp for ChIP DNA.
Antibodies	FANCD2 (Bethyl, A302-174A, lot 1); Brd4 (Bethyl Laboratories A301-985A, lot 5); H3K27ac (Millipore 07–360, lot 2762292).
Peak calling parameters	ChIP-seq reads were trimmed with Cutadapt version 1.18. All reads aligning to the ENCODE hg19 v1 blacklist regions were identified by alignment with BWA version 0.7.17 and removed with Picard SamToFastq, https://broadinstitute.github.io/picard/. Remaining reads were aligned to an hg19 reference genome using BWA. Reads with a mapQ score less than 6 were removed with SAMtools version 1.6 and PCR duplicates were removed with Picard MarkDuplicates. Peaks were called by comparing each ChIP sample to its matching input sample. For FANCD2, the mean fragment size was estimated by Phantompeakqualtools version 2.0. Peaks were called using SICER version 1.1 with the following parameters: redundancy threshold of 100, effective genome fraction of 0.75, window size of 25,000 bp, and gap size of 50,000 bp. H3K27ac and Brd4 peaks were called using macsBroad (macs version 2.1.1 from 2016/03/09) with the following parameters: --broad-cutoff 0.01 -f "BAMPE". Data was converted into bigwigs for viewing and normalized by reads per genomic content (RPGC) using deepTools version 3.0.1 using the following parameters: --binSize 25 --smoothLength 75 --effectiveGenomeSize 2700000000 --centerReads --normalizeUsing RPGC. RPGC-normalized input values were subtracted from RPGC-normalized ChIP values of matching cell type genome-wide using Deeptools with --binSize 25.
Data quality	All ChIP-seq peaks used in this study had an FDR value of ≤0.01. FANCD2 ChIP-seq peaks were filtered by a -log10 q-value of ten or above to remove low-confidence calls, and compared to a previous FANCD2 ChIP-chip dataset using regioneR software that showed strong overlap in called peaks between the datasets (p<0.0001). FANCD2-enriched regions also showed strong correlation with published mitotic DNA synthesis regions, which also mark fragile sites, in the same cell type (p<0.0001). For the Brd4 and H3K27ac datasets, 19% and 40% peaks were above 5-fold enrichment, respectively. Consensus peak sets were used for H3K27ac, defined as overlapping regions found in at least four out of eight W12 ChIP-seq samples. Enhancers were defined as genomic intervals that overlapped between H3K27ac peaks and Brd4 peaks.
Software	See Peak calling parameters for software used in this study for analysis of ChIP-seq data. DiffBind was used to identify consensus H3K27ac peaks. Rank Ordering of Super-Enhancers (ROSE) tool was used to identify super-enhancers using default parameters.
